# Microrheological study on the entanglement dynamics of salt-free polyelectrolyte solutions in the semidilute entangled regime

**DOI:** 10.1038/s41428-025-01079-9

**Published:** 2025-08-15

**Authors:** Atsushi Matsumoto, Ikuto Kato, Chi Zhang, Shinji Sugihara, Yasushi Maeda, Frank Scheffold, Amy Q. Shen

**Affiliations:** 1 Department of Applied Chemistry and Biotechnology, University of Fukui, Fukui-shi, Fukui Japan; 2 Department of Physics, University of Fribourg, Fribourg, Fribourg Switzerland; 3 Micro/Bio/Nanofluidics Unit, Okinawa Institute of Science and Technology Graduate University, Okinawa, Japan

**Keywords:** Polymers, Mechanical properties

## Abstract

We investigate the entanglement dynamics of salt-free aqueous solutions of poly(sodium styrenesulfonate) (NaPSS) across a range of molecular weights, focusing on the semidilute entangled regime. By combining conventional bulk shear rheometry with diffusing wave spectroscopy (DWS) microrheometry, we characterize key parameters, including the entanglement concentration, plateau modulus, reptation time, and Rouse time of entanglement strands. A clear crossover from polyelectrolyte-like to neutral polymer-like behavior is identified as the degree of polymerization decreases, corresponding to a critical monomer concentration *c*_D_ ~ 0.3 M and a critical degree of polymerization N^*^ ~ 6000. The experimental scaling relationships closely agree with the predictions from the Dobrynin model. Our findings provide new insights into the long-standing debate on the entanglement dynamics of polyelectrolytes and establish a framework for analyzing and controlling the solution properties of salt-free polyelectrolytes in the semidilute entangled regime. Moreover, they demonstrate the power of DWS microrheology for probing complex rheological behavior in polyelectrolyte systems.

## Abstract (web summary)

The entanglement dynamics of salt-free aqueous solutions of poly(sodium styrenesulfonate) was investigated using conventional bulk shear rheometry and diffusing wave spectroscopy microrheometry. Our scaling analysis of the entanglement concentration, plateau modulus, and relaxation time revealed a clear crossover from polyelectrolyte-like to neutral polymer-like behavior at a critical monomer concentration *c*_D_ ~ 0.3 M and a critical degree of polymerization N^*^ ~ 6000. We also found that the polymer concentration and molecular weight dependence of the tested entanglement parameters showed good agreement with the Dobrynin model.

## Graphical abstract

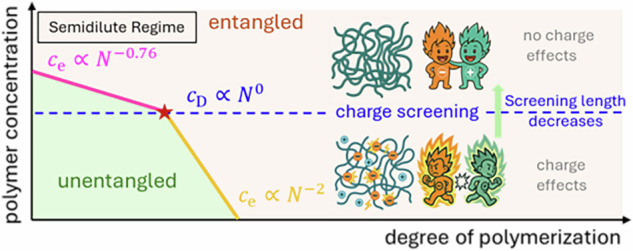

## Introduction

The conformation of polyelectrolyte chains is strongly affected by electrostatic interactions between charged repeating units [1]. Consequently, understanding how these electrostatic interactions alter the dynamics of polyelectrolyte chains is a critical question in polymer physics. Among the existing theoretical frameworks [2–5], Dobrynin’s scaling model has shown good agreement with a wide range of experimental data for polyelectrolyte solutions [6–12]. In their model, the Debye-Hückel theory is employed to describe the electrostatic contributions to the conformation of polyelectrolyte chains, which are then incorporated into viscoelastic models to derive the scaling laws for key rheological parameters, such as solution viscosity and relaxation time [3]. However, recent rheological studies on polyelectrolyte solutions in the semidilute entangled (SE) regime have reported results that challenge the applicability of the traditional tube model developed for entangled polymer systems [8–10, 12–22]. For example, Lopez [18] reported that the dependence of the entanglement concentration on the chain length for salt-free SE polyelectrolyte solutions was significantly weaker than that predicted by Dobrynin’s scaling theory. Moreover, Dobrynin reported that the entanglement concentration was independent of the concentration of added salt. Additionally, Han and Colby [21] reported that the plateau modulus of poly(cesium styrene sulfonate) in glycerol exhibited a scaling consistent with the predicted exponent for electrically neutral polymers in the SE regime. These experimental results suggest the absence of charge effects on the entanglement dynamics of polyelectrolytes in solution. Therefore, independent verification is necessary to understand the entanglement dynamics of polyelectrolytes, given their importance in diverse biological and industrial processes.

The scaling analysis of polyelectrolyte SE solutions presents significant challenges largely because the concentration range of the SE regime, where electrostatic interactions influence the viscoelastic properties of polyelectrolyte solutions, tends to be narrow [23]. According to the Dobrynin model [3], the polyelectrolyte SE regime is defined by the concentration range of *c*_e_ ≤ *c* < *c*_D_, where *c*_e_ is the entanglement concentration at which entanglements start to form and where *c*_D_ is the critical polymer concentration at which the correlation length becomes comparable to the size of an electrostatic blob. When the polymer concentration exceeds this concentration (i.e., *c* > *c*_D_), Dobrynin model predicts the screening of the charge effects, and their viscoelastic properties follow the scaling laws predicted for neutral polymer solutions in the SE regime. Another experimental challenge lies in the gradual nature of the transition between neighboring polymer concentration regimes. As a result, the experimental results with narrow SE regimes may overestimate or underestimate the scaling exponent for the polyelectrolyte SE regime because overlapping contributions from adjacent regimes have different scaling exponents [18]. Furthermore, probing the entanglement dynamics of polyelectrolyte solutions at high frequencies is particularly difficult. In conventional bulk rheometry, the measurable viscoelastic response of polyelectrolyte SE solutions is often limited to the terminal regime at low frequencies since these systems typically utilize low-viscosity solvents, such as water [21]. To assess high frequencies, complex modulus, G^*^, measurements must be performed at various temperatures to construct the master curve of G^*^ by applying the timetemperature superposition (TTS) principle [24]. However, for polyelectrolyte systems, the TTS approach often breaks down due to solvent crystallization during cooling. As a result, most previous studies have been limited to examining the zero-shear viscosity and the longest relaxation time, which restricts the ability to fully test theoretical scaling predictions.

To overcome these experimental challenges, we recently employed a microrheological approach using diffusing wave spectroscopy (DWS) to investigate the entanglement dynamics of aqueous solutions of poly(sodium styrene sulfonate) (NaPSS) with a high molecular weight of *M*_w_ = 3.16 MDa and a narrow molecular weight distribution of *M*_w_/*M*_n_ < 1.35 [25]. DWS is a dynamic light scattering technique tailored for turbid samples that operates in the multiple light scattering regime [26]. DWS-based microrheology enables the measurement of the G^*^ of viscoelastic materials at angular frequencies as large as 10^7^ rad/s, depending on the size and concentration of tracer particles. Over the past two decades, DWS has undergone significant advancements [27–29], allowing more reliable linear rheological measurements across a wide range of soft matter systems, including polymer solutions [30–34], wormlike micellar solutions [35, 36], emulsions [37, 38], suspensions [39], and gels [40]. Using DWS, we successfully captured the rubbery-plateau regime in aqueous SE solutions of the tested NaPSS sample, enabling quantitative analysis of key entanglement parameters, such as the plateau modulus, reptation time, and Rouse time of an entanglement strand. Our measurements were in reasonable agreement with the scaling laws predicted by the Dobrynin model for salt-free polyelectrolyte SE solutions. These results support the conclusion that electrostatic interaction influences the viscoelastic properties of polyelectrolyte solutions in the SE regime.

Building on the successful application of DWS, in this study, we extended our DWS analysis to investigate the entanglement dynamics of NaPSS in water at various molecular weights. Specifically, we aimed to clarify the dependence of *c*_e_ on the degree of polymerization, *N*. We found a transition in the power-law behavior of *c*_e_ from *c*_e_ ∝ *N*^−0.76^ to *c*_e_ ∝ *N*^−2^ at *N* ~ 6000. The transition was also observed in the dependence of the plateau modulus and the relaxation time on the monomer concentration. The observed scaling exponents agreed well with those predicted for neutral polymer and polyelectrolyte solutions, suggesting that the transition marks a shift in the entanglement dynamics of NaPSS from polyelectrolyte-like to neutral polymer-like behaviors. The critical monomer concentration associated with this transition was *c*_D_ ~ 0.3 mol/L, which was in good agreement with the value predicted by the Dobrynin scaling model [23]. Overall, our DWS results demonstrate that short polyelectrolyte chains behave like neutral polymers in the SE regime when *c*_e_ > *c*_D_, the threshold being accurately captured by the Dobrynin model.

## Background theory

**Fig. 1:**
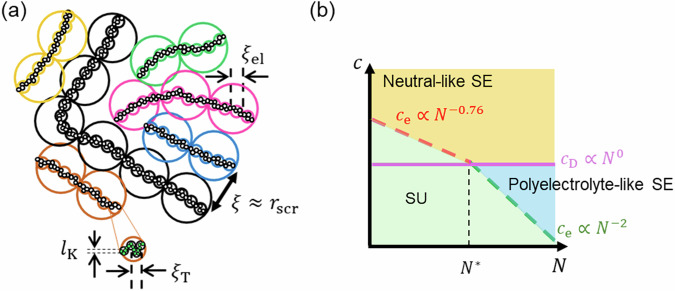
**a** Conformation of a salt-free semidilute polyelectrolyte chain consisting of four blobs of different sizes. **b** Mapping of polymer concentration regimes in a plot of the monomer concentration, *c*, against the degree of polymerization, *N*. The scaling behavior of *c*_e_ changes from *c*_e_ ∝ *N*^−0.76^ to *c*_e_ ∝ *N*^−2^ at a critical condition defined by *c*_D_ and N^*^. The regions highlighted in blue and orange denote the semidilute entangled (SE) regime, in which charge effects on the entanglement dynamics are present and absent, respectively. The green region indicates the semidilute unentangled (SU) regime

In this section, we briefly review the scaling model proposed by Dobrynin et al. [1, 3, 23]. Figure 1a shows a schematic illustration of the conformation of a salt-free semidilute polyelectrolyte chain. In this model, the polyelectrolyte chain is segmented into four different regions, or “blobs”, each characterized by the dominant interactions that govern the local chain conformation within the blob. The electrostatic interaction primarily influences the chain conformation within the region sandwiched between the electrostatic blob and the correlation blob. The size of the electrostatic blob, *ξ*_el_, is predicted as:

1 $${\xi }_{{{{\rm{el}}}}}\approx \left\{\begin{array}{ll}{\left({l}_{{{{\rm{K}}}}}b\right)}^{2/3}{\left(\frac{1}{{l}_{{{{\rm{B}}}}}\,{f}^{2}}\right)}^{1/3}\hfill &{{{\rm{for}}}}\quad T\ll \theta ,\\ {\left({l}_{{{{\rm{K}}}}}b\right)}^{2/3}{\left(\frac{1}{{l}_{{{{\rm{B}}}}}\,{f}^{2}}\right)}^{1/3}\hfill &{{{\rm{for}}}}\quad T=\theta ,\\ {\left({l}_{{{{\rm{K}}}}}b\right)}^{6/7}{\xi }_{{{{\rm{T}}}}}^{-2/7}{\left(\frac{1}{{l}_{{{{\rm{B}}}}}\,{f}^{2}}\right)}^{3/7}\quad &{{{\rm{for}}}}\quad T > \theta ,\end{array}\right.$$

while the size of the correlation blob, *ξ*, is given by

2 $$\xi \left(\approx {r}_{{{{\rm{rsc}}}}}\right)\approx {\left(\frac{B}{cb}\right)}^{\frac{1}{2}}.$$

Under salt-free conditions, *ξ* is equivalent to the electrostatic screening length, *r*_rsc_, up to a prefactor of the order of unity. Here, *l*_K_ is the Kuhn length, *b* is the monomer length, *f* is the charge fraction, *l*_B_ is the Bjerrum length, *c* is the monomer concentration, *θ* is the theta temperature, and *ξ*_T_ is the thermal blob size. The parameter *B* is defined as the ratio of the end-to-end distance of an electrostatic blob to its fully stretched contour length:

3 $$B=\frac{b{g}_{{{{\rm{el}}}}}}{{\xi }_{{{{\rm{el}}}}}}\approx \left\{\begin{array}{ll}{b}^{4/3}{l}_{{{{\rm{K}}}}}^{-2/3}{\left({l}_{{{{\rm{B}}}}}\,{f}^{2}\right)}^{-2/3}\hfill &{{{\rm{for}}}}\quad T\ll \theta ,\\ {b}^{2/3}{l}_{{{{\rm{K}}}}}^{-1/3}{\left({l}_{{{{\rm{B}}}}}\,{f}^{2}\right)}^{-1/3}\hfill &{{{\rm{for}}}}\quad T=\theta ,\\ {b}^{4/7}{l}_{{{{\rm{K}}}}}^{-3/7}{\xi }_{{{{\rm{T}}}}}^{1/7}{\left({l}_{{{{\rm{B}}}}}\,{f}^{2}\right)}^{-2/7}\quad &{{{\rm{for}}}}\quad T > \theta .\end{array}\right.$$

According to Eq. (2), the correlation length decreases with increasing polymer concentration, whereas the electrostatic blob, given by Eq. (1), is independent of the polymer concentration. As a result, the correlation blob overlaps with the electrostatic blob at a critical concentration, *c*_D_. The expression of *c*_D_ is given by

4 $${c}_{{{{\rm{D}}}}}\approx \left\{\begin{array}{ll}\frac{1}{b{l}_{{{{\rm{K}}}}}^{2}}\quad &{{{\rm{for}}}}\quad T\ll \theta ,\\ \frac{1}{{l}_{{{{\rm{K}}}}}^{2/3}bB}\quad &{{{\rm{for}}}}\quad T=\theta ,\\ \frac{1}{b{l}_{{{{\rm{K}}}}}^{2}{B}^{2}}\quad &{{{\rm{for}}}}\quad T > \theta .\end{array}\right.$$

For concentrations *c* > *c*_D_, electrostatic interactions no longer influence the properties of polyelectrolyte solutions. As a result, the entanglement properties of polyelectrolyte solutions resemble those of neutral polymers in good solvents. Therefore, electrostatic interactions influence the entanglement dynamics only when the entanglement concentration *c*_e_ is smaller than *c*_D_.

The entanglement concentration *c*_e_ is derived under the assumption that progressive binary contacts of correlation blobs eventually form an entanglement. The expression of *c*_e_ for salt-free polyelectrolyte solutions is thus given by

5 $${c}_{{{{\rm{e}}}}}={P}_{{{{\rm{e}}}}}^{4}{c}^{* },$$

where *P*_e_ is the packing number, denoting the number of entanglement strands per tube diameter. Accordingly, the scaling law of *c*_e_ with respect to the degree of polymerization, *N*, is derived as

6 $${c}_{{{{\rm{e}}}}}\propto \left\{\begin{array}{ll}{N}^{-0.76}\quad &{{{\rm{for}}}}\,{{{\rm{neutral}}}}\,{{{\rm{polymers}}}}\,{{{\rm{in}}}}\,{{{\rm{good}}}}\,{{{\rm{solvents}}}},\\ {N}^{-2} &\hskip -2pc{{{\rm{for}}}}\,{{{\rm{salt}}}}-{{{\rm{free}}}}\,{{{\rm{polyelectrolytes}}}}.\end{array}\right.$$

Based on Eqs. (4) and (6), we illustrate a boundary between the semidilute unentangled (SU) regime and the semidilute entangled (SE) regime for polyelectrolyte solutions in Fig. 1b. At sufficiently low *c* and *N* values, all the polyelectrolyte solutions lie in the SU regime, where entanglements are absent. As either *c* or *N* increases, the solutions enter the SE regime once the monomer concentration exceeds *c*_e_. A key feature of this phase map is the presence of a second boundary, shown as the horizontal magenta solid line, representing *c*_D_ given by Eq. (4). This line marks the threshold above which the entanglement properties of polyelectrolyte solutions resemble those of neutral polymer solutions. Since *c*_D_ is independent of *N*, it introduces a crossover in the scaling of *c*_e_ against *N*: from *c*_e_ ∝ *N*^−0.76^ for neutral polymers in good solvents to *c*_e_ ∝ *N*^−2^ for salt-free polyelectrolyte solutions. The intersection point of these scaling regimes defines a critical degree of polymerization, N^*^, at which *c*_e_ = *c*_D_. A similar transition in power-law behavior is also expected to be observed for other key entanglement parameters. While the Dobrynin model predicts this transition, it has not been clearly identified in experimental results until now.

Table 1 summarizes the scaling laws of key viscoelastic parameters derived via the Doi-Edwards (DE) tube model for salt-free polyelectrolyte [3] and neutral polymer [41] solutions in the SE regime. Here, the plateau modulus, *G*_N_, the Rouse time of an entanglement strand, *τ*_e_, and the reptation time, *τ*_rep_, are specific only for entangled solutions. Following the approach proposed by Han and Colby [21], we also consider the ratio of *τ*_rep_/*τ*_e_, which is introduced to account for the influence of retarded solvent dynamics on the polymer relaxation times. These scaling laws serve as the basis for comparison with our DWS microrheology results.

**Table 1:** Comparison of the scaling laws of the entanglement concentration, *c*_e_, the specific viscosity, *η*_sp_, the plateau modulus, *G*_e_, the Rouse time of an entanglement strand, *τ*_e_, and the reptation time, *τ*_rep_, for salt-free polyelectrolyte solutions [3] and neutral polymers [41] in good solvents

Semidilute Entangled (SE) regime			
	Salt-free polyelectrolytes	Neutral polymers	Our results
*c*_e_	*N*^−2^	*N*^−0.76^	*N*^−0.76^ → *N*^−2^^a^
*η*_sp_	*N*^3^*c*^1.5^	*N*^3^*c*^3.9^	–
*G*_N_	*N*^0^*c*^1.5^	*N*^0^*c*^2.3^	*c*^1.1^ → *c*^2.0^^b^
*τ*_e_	*N*^0^*c*^−1.5^	*N*^0^*c*^−2.3^	–
*τ*_rep_	*N*^3^*c*^0^	*N*^3^*c*^1.6^	–
*τ*_rep_/*τ*_e_	*N*^3^*c*^1.5^	*N*^3^*c*^3.9^	*c*^1.2^ → *c*^3.1^^b^

More details on the scaling law can be found in the original article by Dobrynin et al. [3, 23]

^a^The arrow indicates the transition in the power-law behavior as the molar mass of NaPSS increases. Note that the scaling exponent carries uncertainty in its magnitude, as it is estimated based on a curve fit to only two data points

^b^The arrow indicates the transition in the power-law behavior as the polymer concentration of NaPSS increases

## Materials & methods

### Materials and sample preparation

Standard sodium polystyrene sulfonate (NaPSS) polyelectrolytes with various molecular weights were purchased from Polymer Standard Services (*M*_w_ = 3.16 MDa) and Scientific Polymer Products (*M*_w_ = 2.24, 1.21, 0.786 MDa). Prior to use, all samples were dialyzed against deionized (DI) water to remove ionic impurities and then recovered in powder form via a freeze-drying method. Dialysis was performed using a dialysis tube with a nominal molecular weight cutoff of 12,000–14,000. During the dialysis process, the solvent was replaced with fresh water twice a day until the ionic conductivity of the solvent fell below the ambient level of 4 μS cm^−1^. DI water was produced via a water purification system (ICW-3000, Merck) and used as the solvent. For DWS experiments, a 5 wt% suspension of monodisperse polystyrene (PS) particles 211 ± 8 nm in diameter was purchased from microParticles GmbH and used as received.

Test solutions for bulk shear rheology and DWS microrheology experiments were prepared by directly dissolving NaPSS in DI water at room temperature. For DWS measurements, the particle concentration was adjusted to 0.7 wt% by diluting the NaPSS solutions with a 5 wt% PS particle stock suspension. A homogeneous dispersion of PS particles in NaPSS solutions was achieved by vortexing the mixture at room temperature. DWS experiments were conducted only when the particles were uniformly dispersed throughout the solution without visible aggregation. As a result, because of the elevated viscosity and increased ionic strength of NaPSS solutions at high *c*, our scaling analysis based on DWS cannot be extended to the high-*c* regime, where stable dispersions cannot be reliably maintained.

### Bulk shear rheometry

The shear viscosity, *η*, at 25 °C was measured via a stress-controlled rheometer (MCR702e, Anton Paar) by varying the shear rate $\dot{\gamma }$ from 0.01 to 1000 s^−1^. A stainless steel cone plate with a diameter of 50 mm and a cone angle of 1° was used as the upper geometry, whereas a stainless steel parallel plate with a diameter of 60 mm served as the lower geometry. The measurement temperature was controlled via a Peltier system (P-PTD200, Anton Paar), and a solvent trap was used to prevent sample evaporation.

The same rheometer setup, including the fixtures and solvent trap, was used to measure the complex modulus, ${G}_{{{{\rm{bulk}}}}}^{* }$, at 25 °C in the frequency range of 0.1 ≤ *ω* ≤ 100 rad s^−1^. The shear strain, *γ*, was set at 10% to ensure the linear regime where the value of ${G}_{{{{\rm{bulk}}}}}^{* }$ is independent of *γ*.

### Diffusing wave spectroscopy

DWS measurements were performed via a commercially available apparatus (DWS RheoLab, LS Instruments) with transmitted light of 685 nm in wavelength. Using software provided by the manufacturer, the complex modulus, ${G}_{{{{\rm{DWS}}}}}^{* }$, was estimated from the intensity autocorrelation function data [28]. Briefly, the DWS apparatus acquires the photon correlation function in a common multi-tau mode in which the linearly arranged data points are grouped and spaced logarithmically [42, 43]. Except for a few data points omitted by truncation, each autocorrelation function data point was converted into a value of mean square displacement, $< \Delta {r}^{2}\left(t\right) >$, and subsequently a value of the storage modulus, ${G}_{{{{\rm{DWS}}}}}^{{\prime} }$, and loss modulus, ${G}_{{{{\rm{DWS}}}}}^{{\prime\prime} }$. More details about the measurement principle of DWS can be found elsewhere [26, 40]. In our DWS experiments, the multi-tau duration was set at 120 s. To estimate the $< \Delta {r}^{2}\left(t\right) >$ values, the refractive indices of the NaPSS solutions were measured via a refractometer (Abbemat MW, Anton Paar) at 632.8 nm, with the assumption that the refractive index difference at 685 nm and 632.8 nm is negligible.

**Fig. 2:**
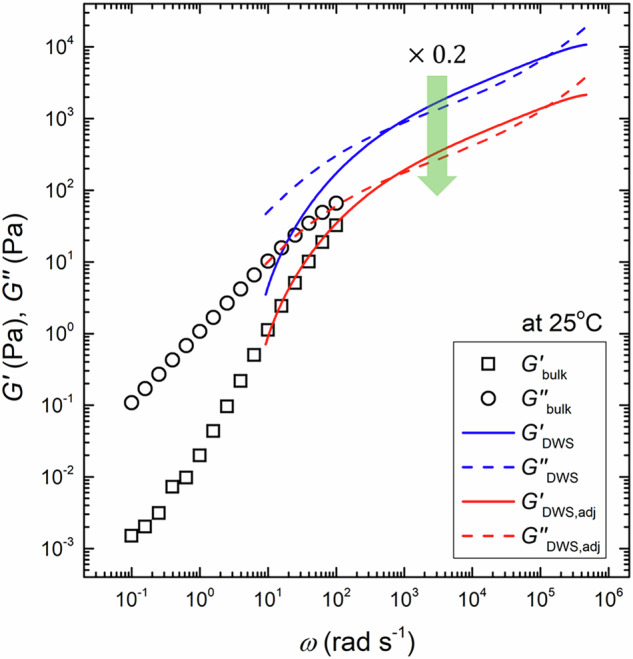
DWS data are processed by shifting the measured ${G}_{{{{\rm{DWS}}}}}^{* }$ vertically to align with the bulk rheology data, i.e., ${G}_{{{{\rm{bulk}}}}}^{* }$. The adjusted DWS spectra are denoted as ${G}_{{{{\rm{DWS}}}},{{{\rm{adj}}}}}^{* }$. Square symbols represent the measured bulk complex modulus, ${G}_{{{{\rm{bulk}}}}}^{* }$. The blue and red lines correspond, respectively, to the original complex modulus, ${G}_{{{{\rm{DWS}}}}}^{* }$, and the corrected complex modulus, ${G}_{{{{\rm{DWS}}}},{{{\rm{adj}}}}}^{* }$, obtained via DWS measurements. The solid and dashed lines denote the storage and loss moduli, respectively

In principle, ${G}_{{{{\rm{DWS}}}}}^{* }$ should coincide with ${G}_{{{{\rm{bulk}}}}}^{* }$ obtained from bulk rheometry. However, in reality, discrepancies are often observed between ${G}_{{{{\rm{DWS}}}}}^{* }$ and ${G}_{{{{\rm{bulk}}}}}^{* }$ due to the partial aggregation of tracer particles and their interactions with the surrounding medium. Consequently, a correction is often required when reference data from bulk rheometers are used. Figure 2 compares the frequency dependence of ${G}_{{{{\rm{bulk}}}}}^{* }$ for an entangled solution of NaPSS with *M*_w_ = 2.24 MDa at *c* = 0.398 mol/L. We found that ${G}_{{{{\rm{DWS}}}}}^{* }$ was always greater than ${G}_{{{{\rm{bulk}}}}}^{* }$ in the frequency range of 10^1^ < *ω* < 10^3^ rad s^−1^. The same discrepancy was also observed in our previous study [25] and could be attributed to the aggregation of tracer particles caused by electrostatic screening effects from charged polymer chains and dissociated counterions. Such aggregation reduces the $< \Delta {r}^{2}\left(t\right) >$ of tracer particles, which in turn leads to an overestimation of the complex modulus [26]. To correct the observed difference in magnitude between ${G}_{{{{\rm{DWS}}}}}^{* }$ and ${G}_{{{{\rm{bulk}}}}}^{* }$, we applied a vertical shift to the ${G}_{{{{\rm{DWS}}}}}^{* }$ spectra such that the adjusted spectra, denoted as ${G}_{{{{\rm{DWS}}}},{{{\rm{adj}}}}}^{* }$, overlapped with the corresponding ${G}_{{{{\rm{bulk}}}}}^{* }$ spectra. The correction was applied to all the ${G}_{{{{\rm{DWS}}}}}^{* }$ data in this study. The shift factor varied within a range from 0.1 to 0.3 and tended to decrease with increasing *c*, likely due to increased ionic strength at higher polyelectrolyte concentrations. The particle aggregation may introduce the polydispersity of the tracer particles. In this case, the plateau region may be extended due to the broadening of the decay of the intensity autocorrelation function, which likely affects the value of scaling exponents, particularly for relaxation times. However, as we discuss later, we confirmed that the impact of polydispersity was minimal, as the obtained scaling exponent showed reasonable agreement with the predicted scaling exponent. In our previous work, we confirmed that the entanglement parameters extracted from the ${G}_{{{{\rm{DWS}}}},{{{\rm{adj}}}}}^{* }$ spectra were not significantly affected by bead inertia effects or tracer particle size [25].

## Results and discussion

### Specific viscosity and polymer concentration regimes

**Fig. 3:**
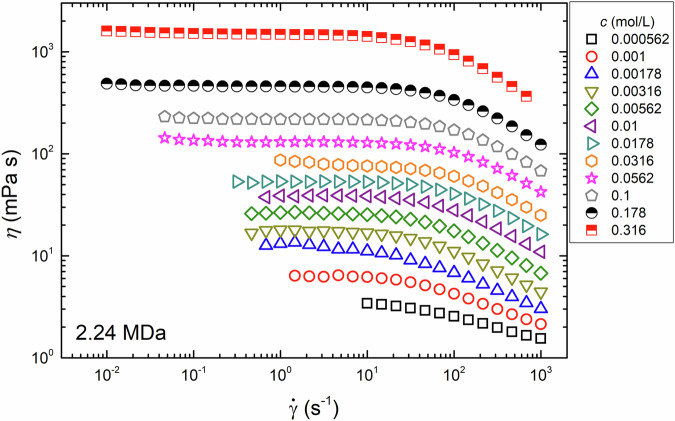
A typical shear viscosity curve is observed in aqueous solutions of NaPSS with *M*_w_ = 2.24 MDa. In this figure, the measured shear viscosity, *η*, at 25 ^∘^C is plotted as a function of the shear rate, $\dot{\gamma }$, for the NaPSS solutions at various monomer concentrations, *c*

We first identified a transition zone from the SU to SE regimes for the tested NaPSS to determine the polymer concentrations to be tested in the DWS experiment. To do so, we measured the steady shear viscosity, *η*, of NaPSS solutions at 25 °C while varying the monomer concentration, *c*, of NaPSS. Figure 3 shows the dependence of *η* on the shear rate, $\dot{\gamma }$, for aqueous solutions of NaPSS with *M*_w_ 2.24 MDa. The value of *η* increased monotonically with increasing *c*. At a fixed *c*, *η* remained constant at low $\dot{\gamma }$ and started to decrease above a critical shear rate, demonstrating typical shear-thinning behavior for polymer solutions [44]. A similar trend of *η* with increasing *c* and $\dot{\gamma }$ was observed for other NaPSS at different *M*_w_ values (see Supplementary Fig. S1 of the supporting information), which is also consistent with the results obtained in our previous study [25].

**Fig. 4:**
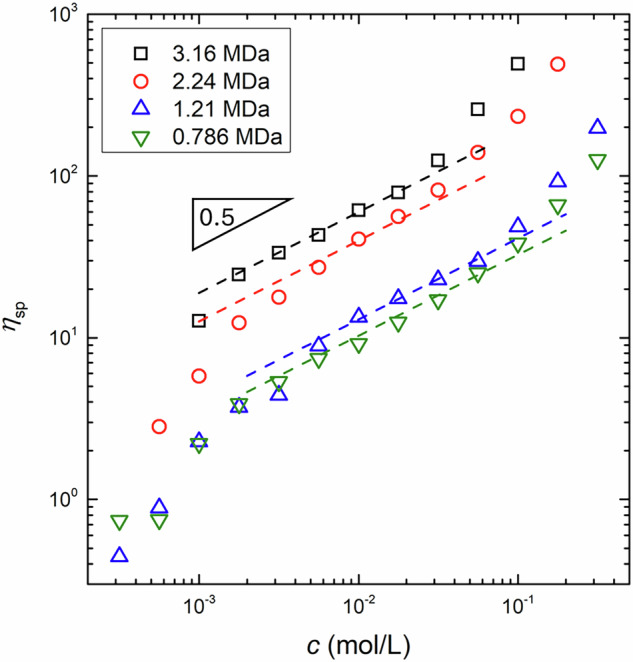
The transition zone from the semidilute unentangled to semidilute entangled regimes is determined as a monomer concentration, *c*, where the scaling of the specific viscosity, *η*_sp_, against *c* deviates from *η*_sp_ ∝ *c*^0.5^ (dashed lines) at high *c*. In this figure, the measured *η*_sp_ at 25 ^∘^C is plotted as a function of *c* for aqueous solutions of NaPSS with molecular weights of 3.16 MDa (black squares), 2.24 MDa (red circles), 1.21 MDa (blue triangles), and 0.786 MDa (green inverted triangles)

The polymer concentration regime was determined through scaling analysis of the specific viscosity, *η*_sp_, with respect to *c*. The specific viscosity is defined as:

7 $${\eta }_{{{{\rm{sp}}}}}\equiv \frac{{\eta }_{0}-{\eta }_{{{{\rm{s}}}}}}{{\eta }_{{{{\rm{s}}}}}},$$

where *η*_0_ and *η*_s_ are the zero-shear viscosities of the polymer solution and the solvent, respectively. The values of *η*_0_ and *η*_s_ were estimated by averaging the *η* values in the Newtonian regime, where *η* was independent of $\dot{\gamma }$. Figure 4 displays the dependence of the estimated *η*_sp_ on *c* for aqueous solutions of four NaPSS with different *M*_w_ values. The value of *η*_sp_ increased sharply with increasing *c* for *c* < 2 × 10^−3^ mol/L, above which the rate of increase in *η*_sp_ slowed until reaching a critical monomer concentration *c*_t_. Notably, the value of *c*_t_ shifted to a higher *c* as *M*_w_ decreased. For *c* > *c*_t_, *η*_sp_ exhibited a stronger dependence on *c* once again. These changes in the slope of *η*_sp_ with respect to *c* may reflect transitions between different polymer concentration regimes. In particular, we observed a scaling of *η*_sp_ ∝ *c*^0.5^ in the intermediate concentration range, which is in good agreement with the scaling predicted for polyelectrolyte solutions in the SU regime. Therefore, the observed stronger scaling behavior at low and high *c* can be attributed to a transition to the dilute and SE regimes, respectively. However, the presence of entanglements at *c* > *c*_t_ cannot be judged solely based on the shear viscosity data. We therefore examined the frequency dependence of the complex modulus for NaPSS solutions at *c* > *c*_t_ via DWS to further investigate the presence of entanglements.

### Overview of the ${G}_{{{{\rm{DWS}}}},{{{\rm{adj}}}}}^{* }$ spectra

**Fig. 5:**
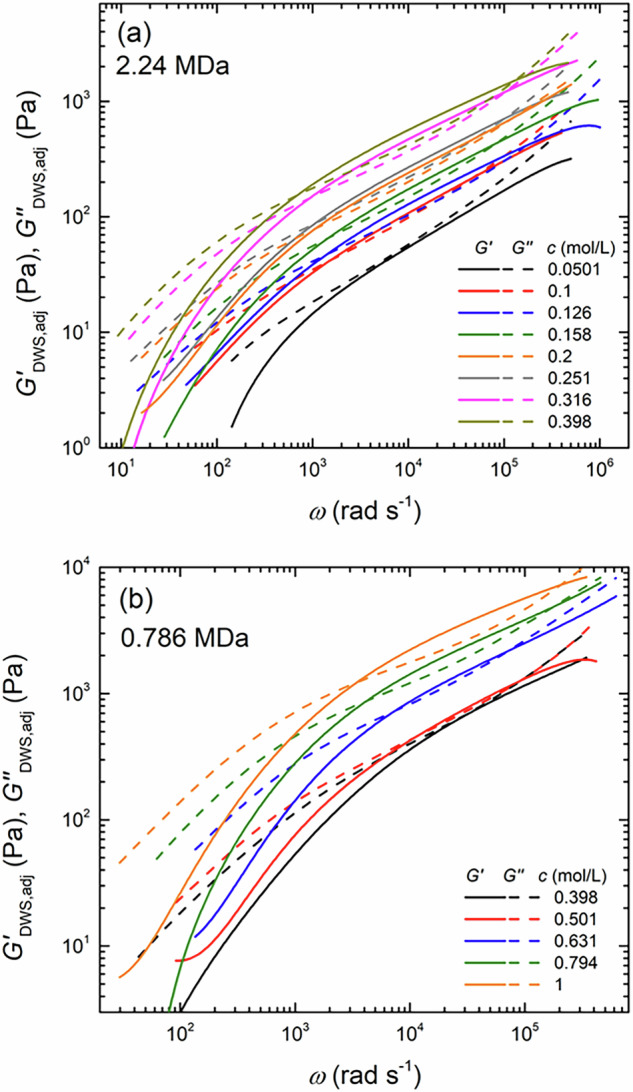
The dependence of the adjusted complex modulus, ${G}_{{{{\rm{DWS}}}},{{{\rm{adj}}}}}^{* }$, on the angular frequency, *ω*, for aqueous solutions of two NaPSS with different molecular weights at **a** *M*_w_ = 2.24 MDa and **b** *M*_w_ = 0.786 MDa, obtained by DWS measurement while varying the monomer concentration, *c*, of NaPSS. The solid and dashed lines denote the storage and loss moduli, respectively

Figure 5 shows the dependence of ${G}_{{{{\rm{DWS}}}},{{{\rm{adj}}}}}^{* }$ on *ω* for aqueous solutions of two NaPSS with high (*M*_w_ = 2.24 MDa) and low (*M*_w_ = 0.786 MDa) molecular weights. For the high-*M*_w_ NaPSS at *c* = 0.0501 mol/L, the loss modulus, ${G}_{{{{\rm{DWS}}}},{{{\rm{adj}}}}}^{{\prime\prime} }$, was always greater than the storage modulus, ${G}_{{{{\rm{DWS}}}},{{{\rm{adj}}}}}^{{\prime} }$, across the entire frequency range, indicating that NaPSS chains do not form entanglements at this monomer concentration. However, when the monomer concentration was elevated to *c* = 0.1 mol/L, ${G}_{{{{\rm{DWS}}}},{{{\rm{adj}}}}}^{{\prime} }$ and ${G}_{{{{\rm{DWS}}}},{{{\rm{adj}}}}}^{{\prime\prime} }$ crossed twice at high and low *ω*, indicating the onset of entanglement among the NaPSS chains. Based on this transition, the entanglement concentration, *c*_e_, was estimated to be approximately *c*_e_ = 0.075 mol/L by taking the midpoint between the two concentrations where unentangled and entangled behaviors were observed. As the monomer concentration increased further, the distance between the two intersection points of ${G}_{{{{\rm{DWS}}}},{{{\rm{adj}}}}}^{{\prime} }$ and ${G}_{{{{\rm{DWS}}}},{{{\rm{adj}}}}}^{{\prime\prime} }$ widened, suggesting a progressive development of entanglement density with increasing *c*. A similar variation in ${G}_{{{{\rm{DWS}}}},{{{\rm{adj}}}}}^{{\prime} }$ with increasing *c* was observed for the low-*M*_w_ NaPSS sample (see Fig. 5b). In contrast, the onset of entanglement formation for the low-*M*_w_ NaPSS was observed at higher *c*, and the value of *c*_e_ was estimated to be *c* = 0.45 mol/L.

Table 2 summarizes the entanglement concentration for each NaPSS examined in this study. Here, the ${G}_{{{{\rm{DWS}}}},{{{\rm{adj}}}}}^{* }$ data for NaPSS at *M*_w_ = 3.16 MDa and *M*_w_ = 1.27 MDa are provided in Supplementary Fig. S2 of the supporting information. Table 2 shows that the entanglement concentration decreased monotonically with increasing molecular weight of NaPSS, which is consistent with the trend predicted by the Dobrynin model. These findings demonstrate that DWS can be used as a powerful tool to probe the entanglement dynamics of polyelectrolyte solutions.

**Table 2:** The weight-averaged molecular weight, *M*_w_, the degree of polymerization, *N*, and the entanglement concentration, *c*_e_, for the NaPSS samples used in this study

*M*_w_ (MDa)	*N*	*c*_e_ (mol/L)
3.16	1.53 × 10^4^	0.045
2.24	1.09 × 10^4^	0.075
1.27	5.85 × 10^3^	0.28
0.786	3.81 × 10^3^	0.45

### Scaling analysis of entanglement parameters

**Fig. 6:**
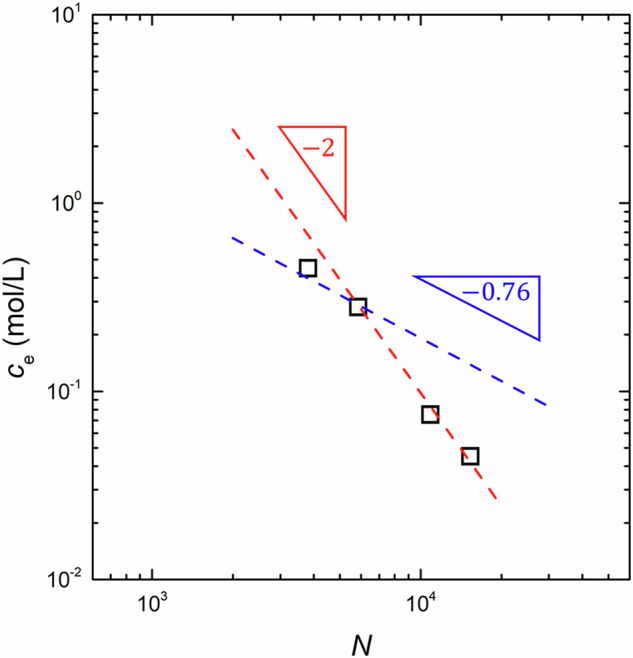
The dependence of the entanglement concentration, *c*_e_, on the degree of polymerization, *N*, for NaPSS in water at 25 °C. The red and blue dashed lines represent the predicted scaling for polyelectrolyte solutions and neutral polymers in good solvents, given by *c*_e_ ∝ *N*^−2^ and *c*_e_ ∝ *N*^−0.76^, respectively

We now perform a scaling analysis of key entanglement parameters via the DWS data. Figure 6 shows the dependence of the obtained *c*_e_ on the degree of polymerization, *N*, of NaPSS. The value of *N* was calculated by dividing *M*_w_ by the molar mass of the NaPSS repeating units, *M*_0_ = 206.19 g/mol, under the assumption that all counterions are associated with the polymer chains. As shown in Fig. 6, for *N* > 6000, the value of *c*_e_ increased linearly with decreasing *N*, following a scaling of *c*_e_ ∝ *N*^−2^. The obtained scaling exponent was consistent with the predicted exponent for polyelectrolyte solutions. However, for *N* < 6000, even though only one data point is available, a deviation from the scaling of *c*_e_ ∝ *N*^−2^ was observed. This suggests that entangled NaPSS chains with *N* < 6000 behave similar to neutral polymers, likely due to sufficient screening of electrostatic interactions. Indeed, the measured dependence of *c*_e_ on *N* for *N* < 6000 aligned with the scaling prediction for neutral polymers in good solvents, given by *c*_e_ ∝ *N*^−0.76^. Based on these observations, the critical monomer concentration, *c*_D_, was estimated as the crossover entanglement concentration between the two power-law regimes, yielding *c*_D_ ~ 0.3 mol/L for NaPSS in water at 25 °C. Notably, the obtained *c*_D_ value closely matches the value of *c*_D_ = 0.28 mol/L reported by Dobrynin and Jacobs [23], which further supports the observed shift in the scaling behavior at *c*_e_ ~ 0.3 mol/L. As a result, the scaling analysis of *c*_e_ demonstrates that electrostatic interactions influence the entanglement properties only for NaPSS with *N* > 6000 at *c* < 0.3 mol/L.

**Fig. 7:**
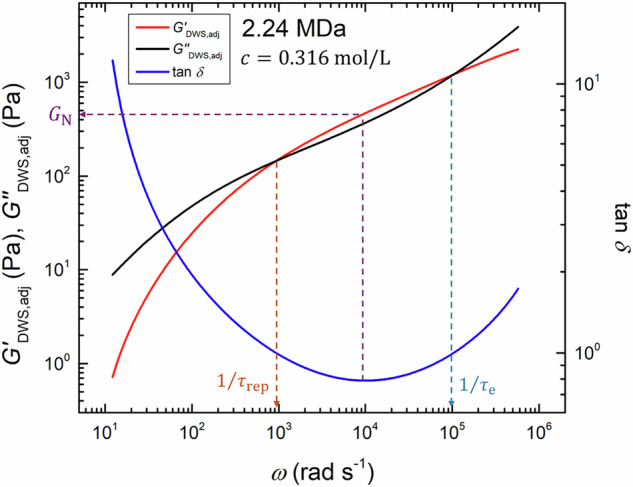
The dashed lines explain how to estimate the plateau modulus, *G*_N_, reptation time, *τ*_rep_, and Rouse time of an entanglement strand, *τ*_e_, from the ${G}_{{{{\rm{DWS}}}},{{{\rm{adj}}}}}^{* }$ spectra. The ${G}_{{{{\rm{DWS}}}},{{{\rm{adj}}}}}^{* }$ data for NaPSS with *M*_w_ = 2.24 MDa at *c* = 0.316 mol/L are used as reference spectra

To test whether this scenario is valid, we evaluated the plateau modulus, *G*_N_, reptation time, *τ*_rep_, and Rouse time of an entanglement strand, *τ*_e_, from the DWS data. Figure 7 illustrates the method used to estimate the values of *G*_N_, *τ*_rep_, and *τ*_e_ from the ${G}_{{{{\rm{DWS}}}},{{{\rm{adj}}}}}^{* }$ spectra. Specifically, the value of *G*_N_ was determined as the ${G}_{{{{\rm{DWS}}}},{{{\rm{adj}}}}}^{{\prime} }$ value at a frequency where the loss tangent, $\tan \delta =\frac{{G}_{{{{\rm{DWS}}}},{{{\rm{adj}}}}}^{{\prime\prime} }}{{G}_{{{{\rm{DWS}}}},{{{\rm{adj}}}}}^{{\prime} }}$, displays a minimum, i.e., based on the MIN method [45]. Here, we emphasize that the choice of the estimation method of *G*_N_ does not influence the scaling analysis presented below, which was confirmed in our previous study [25]. However, the values of *τ*_rep_ and *τ*_e_ were estimated as the inverse of the crossover frequency of ${G}_{{{{\rm{DWS}}}},{{{\rm{adj}}}}}^{{\prime} }$ and ${G}_{{{{\rm{DWS}}}},{{{\rm{adj}}}}}^{{\prime\prime} }$, respectively. The extracted values of *G*_N_, *τ*_rep_, and *τ*_e_ for each NaPSS sample are summarized in Supplementary Table S1 of the supporting information.

**Fig. 8:**
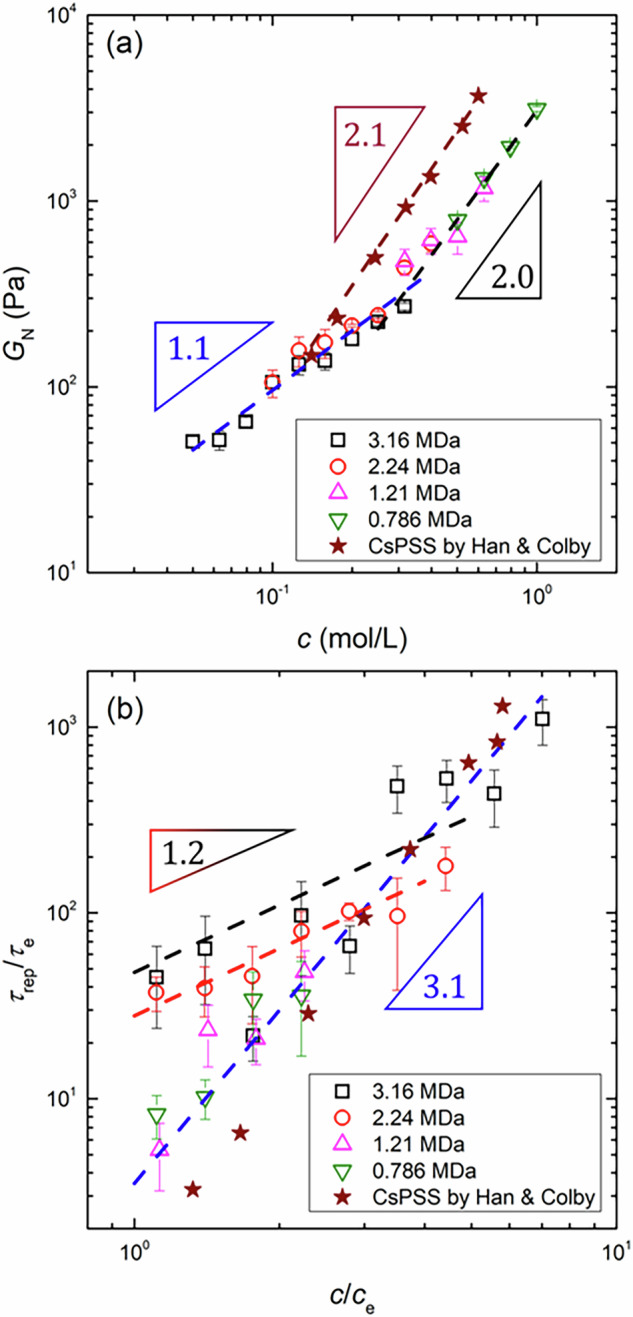
A transition of the power-law behavior is observed in the dependence of (**a**) the plateau modulus, *G*_N_, and (**b**) the relaxation time ratio, *τ*_rep_/*τ*_e_, on the monomer concentration, *c*, of NaPSS. In (**b**), *c* is normalized by the entanglement concentration, *c*_e_, to account for differences in the onset concentration of entanglement formation among the NaPSS samples. The different symbols represent the *G*_N_ and *τ*_rep_/*τ*_e_ data for the NaPSS system with different *M*_w_ values, whereas the dashed lines indicate the power-law fit to the experimental data. The star symbols are the *G*_N_ and *τ*_rep_/*τ*_e_ data for CsPSS in glycerol, reported by Han and Colby [21]. The experimental error at each concentration was obtained from at least five independent measurements

Figure 8a shows the dependence of the estimated *G*_N_ on *c* for the tested NaPSS with various *M*_w_ values. The value of *G*_N_ increased monotonically with increasing *c*, exhibiting two distinct power-law regimes with respect to *c*. At lower *c*, *G*_N_ scaled as *G*_N_ ∝ *c*^1.1^, whereas at higher *c*, the scaling shifted to *G*_N_ ∝ *c*^2.0^. These scaling exponents in the low- and high-*c* regimes were in fair agreement with the predicted values for the polyelectrolyte and neutral polymer solutions, respectively. Therefore, the observed shift in the power-law behavior suggests a screening of charge effects on the entanglement dynamics. Notably, we found that the transition between the two power-law regimes occurred at *c* ~ 0.3 mol/L, which is consistent with the *c*_D_ value estimated from the scaling analysis for *c*_e_ discussed above. Importantly, the polyelectrolyte scaling behavior was observed only in samples with *N* > 6000. Interestingly, two data points at the highest *c* for the NaPSS sample with *M*_w_ = 2.24 MDa (i.e., *N* > 6000) followed the scaling of the neutral polymer solutions. This can be attributed to the fact that these concentrations exceed the threshold of *c*_D_ = 0.3 mol/L, where electrostatic interactions are sufficiently screened. Thus, our scaling analysis for *G*_N_ supports the conclusion drawn from the scaling of *c*_e_ vs. *N*, namely, that the electrostatic interactions influence the entanglement properties only for NaPSS with *N* > 6000 at concentrations below 0.3 mol/L.

A similar transition of the power-law behavior was also observed in the trend of the relaxation time with respect to *c*, as shown in Fig. 8b. Here, by adopting the approach proposed by Han and Colby [21], we plotted the relaxation time ratio of *τ*_rep_/*τ*_e_ to mitigate the potential effects of retarded solvent dynamics on the polymer relaxation times. In addition, the monomer concentration was normalized by the entanglement concentration to account for differences in the onset concentration of entanglement formation among the NaPSS samples. For the NaPSS samples with *M*_w_ = 1.21 and 0.786 MDa (i.e., *N* < 6000), the value of *τ*_rep_/*τ*_e_ followed a scaling of *τ*_rep_/*τ*_e_ ∝ *c*^3.1^ over the entire measured *c* range. In contrast, for the NaPSS samples with *M*_w_ = 2.24 and 3.16 MDa (i.e., *N* > 6000), a shift in the power-law behavior was observed across a critical concentration ratio, ${(c/{c}_{{{{\rm{e}}}}})}^{* }$, from *τ*_rep_/*τ*_e_ ∝ *c*^1.2^ to *τ*_rep_/*τ*_e_ ∝ *c*^3.1^. These scaling exponents in the low- and high-*c* regimes were in reasonable agreement with the predicted values for the polyelectrolyte and neutral polymer solutions, respectively. Therefore, the observed shift in the power-law behavior reflects a screening of charge effects on the entanglement dynamics. The value of ${(c/{c}_{{{{\rm{e}}}}})}^{* }$ was found to depend on the molecular weight of NaPSS, with values of ${(c/{c}_{{{{\rm{e}}}}})}^{* } \sim 4$ for *M*_w_ = 3.16 MDa and ${(c/{c}_{{{{\rm{e}}}}})}^{* } \sim 3$ for *M*_w_ = 2.24 MDa. Thus, by multiplying the entanglement concentration for each NaPSS, the critical monomer concentration was estimated to be *c*_D_ ~ 0.2 mol/L, independent of the molecular weight of NaPSS. The obtained *c*_D_ was consistent with the values obtained from the scaling analysis of both *c*_e_ and *G*_N_. Furthermore, the observed *M*_w_-independent behavior of *c*_D_ agreed with the scaling prediction for *c*_D_ by the Dobrynin model [3]. Overall, our DWS analysis of entanglement dynamics revealed the existence of a critical polymer concentration above which the charge effects are screened, the system behaves like a neutral polymer solution, and its concentration value can be predicted via the Dobrynin model, given by Eq. (4). The reported discrepancies between experimental results and theoretical predictions may stem from the fact that *c*_e_ of the tested polyelectrolytes exceeds *c*_D_ under the experimental conditions. These results highlight the importance of identifying the value of *c*_D_ for the polyelectrolytes of interest to accurately predict their entanglement properties in solution.

### Comparison with literature bulk rheology data

We further validated our DWS results by comparing them with literature data obtained via conventional bulk rheometers. Figure 8 includes the *G*_N_ and *τ*_rep_/*τ*_e_ data for poly(cesium styrene sulfonate) (CsPSS) dissolved in glycerol, reported by Han and Colby [21]. Since glycerol is a glass-forming solvent, the rubbery-plateau regime was detected by constructing the master curve of the measured ${G}_{{{{\rm{bulk}}}}}^{* }$ at various temperatures. The entanglement concentration was estimated as *c*_e_ ~ 0.2 mol/L via the same methodology described above. As discussed in their study, both *G*_N_ and *τ*_rep_/*τ*_e_ followed the scaling predicted for SE solutions of neutral polymers. Remarkably, the literature data closely overlapped with our DWS results, supporting the reliability and consistency of our DWS analysis.

Notably, the measured *G*_N_ for CsPSS in glycerol intersected with the scaling line of *G*_N_ ∝ *c*^1.1^ for NaPSS in water at *c* = 0.126 mol/L. This crossover point likely corresponds to the critical monomer concentration *c*_D_ in glycerol. Here, we recall the scaling law of *c*_D_, given by Eq. (4). Assuming that the Manning prediction holds for the charge fraction, i.e., *f* ∝ *ε*_r_, the relationship between *c*_D_ and the solvent dielectric constant *ε*_r_ can be expressed as ${c}_{{{{\rm{D}}}}}\propto {\varepsilon }_{{{{\rm{r}}}}}^{4/7}$ in good solvents [46]. Given that *ε*_r_ = 81 for water and *ε*_r_ = 42 for glycerol, it is reasonable to observe a smaller *c*_D_ value in glycerol than in water. Indeed, the Dobrynin model predicts a critical monomer concentration of *c*_D_ ~ 0.2 mol/L in glycerol at 25 °C, which is in reasonable agreement with the observed crossover point.

## Conclusion

In this study, we systematically investigated the entanglement dynamics of NaPSS in water across a range of molecular weights while focusing on the properties in the SE regime. By using both classical bulk shear rheology and DWS microrheology techniques, we probed the viscoelastic properties of salt-free SE solutions of NaPSS in detail. We first determined the transition zone from the SU to the SE regimes for each tested NaPSS by measuring *η*_sp_ at various *c* values. After that, we measured the complex modulus ${G}_{{{{\rm{DWS}}}}}^{* }$ using DWS and successfully observed the rubbery-plateau regime in aqueous SE solutions of NaPSS. From these spectra, we extracted key entanglement parameters, including *c*_e_, *G*_N_, *τ*_rep_, and *τ*_e_. A plot of *c*_e_ as a function of *N* revealed a transition in the power-law behavior from *c*_e_ ∝ *N*^−0.76^ to *c*_e_ ∝ *N*^−2^. The obtained scaling exponents agreed with the predicted values for the neutral polymer and polyelectrolyte solutions. Therefore, this transition signifies a shift in the entanglement dynamics of NaPSS chains from polyelectrolyte-like to neutral polymer-like behavior. The critical monomer concentration at which this transition occurred was estimated as *c*_D_ ~ 0.3 mol/L for NaPSS in water at 25 °C, which is consistent with the value predicted by the Dobrynin scaling model. The corresponding critical degree of polymerization was identified as N^*^ ~ 6000 at 25 °C. Consistent scaling transitions were also observed in the dependence of *G*_N_ and *τ*_rep_/*τ*_e_ on *c*, further reinforcing the presence of a well-defined boundary between the polyelectrolyte-like and neutral-like entanglement regimes. This boundary condition was captured well by the Dobrynin model. Overall, our DWS-based microrheological analysis demonstrated that the long-standing debate on the entanglement dynamics of PEs arises from the screening of electrostatic interactions in the semidilute entangled regime. By identifying *c*_D_ for a given polyelectrolyte, we enable accurate prediction of the entanglement properties of polyelectrolyte solutions. Beyond resolving previous discrepancies in the literature, our results establish DWS microrheology as a powerful tool for probing entanglement dynamics in polyelectrolyte systems, particularly in regimes inaccessible to conventional rheometry. This work not only clarifies the fundamental physics governing polyelectrolyte behavior in the semidilute entangled regime but also provides a framework for designing polyelectrolyte solutions with tailored viscoelastic properties for applications in fields such as biomaterials, coatings, and soft electronics. Future studies may build on this foundation to explore the role of solvent quality, additive salts, and multivalent ions in modulating entanglement dynamics.

## Supplementary information

Supporting information
