# Supplementary material for: Microrheological study on the entanglement dynamics of salt-free polyelectrolyte solutions in the semidilute entangled regime
Source: Polym J. 2025 Aug 15;57(11):1215–25. doi: 10.1038/s41428-025-01079-9 (PMC12586176; doi:10.1038/s41428-025-01079-9)
Supplement: Supplementary file 1 — Supporting information [file 41428_2025_1079_MOESM1_ESM.pdf]

Supporting Information: Microrheological Study on Entanglement Dynamics of Salt-Free Polyelectrolyte Solutions in the Semidilute Entangled Regime

Atsushi Matsumoto,*,† Ikuto Kato,† Chi Zhang,‡ Shinji Sugihara,† Yasushi Maeda,† Frank Scheffold,‡ and Amy Q. Shen¶

†*Department of Applied Chemistry and Biotechnology, University of Fukui, 3-9-1 Bunkyo, Fukui-shi, 910-8507 Fukui, Japan*

‡*Department of Physics, University of Fribourg, Chemin du Musée 3, Fribourg, 1700, Fribourg, Switzerland*

¶*Micro/Bio/Nanofluidics Unit, Okinawa Institute of Science and Technology Graduate University, 1919-1 Tancha, Onna-son, Kunigami-gun, 904-0495 Okinawa, Japan*

E-mail: atsushi5@u-fukui.ac.jp

S1. Supporting Figures & Tables

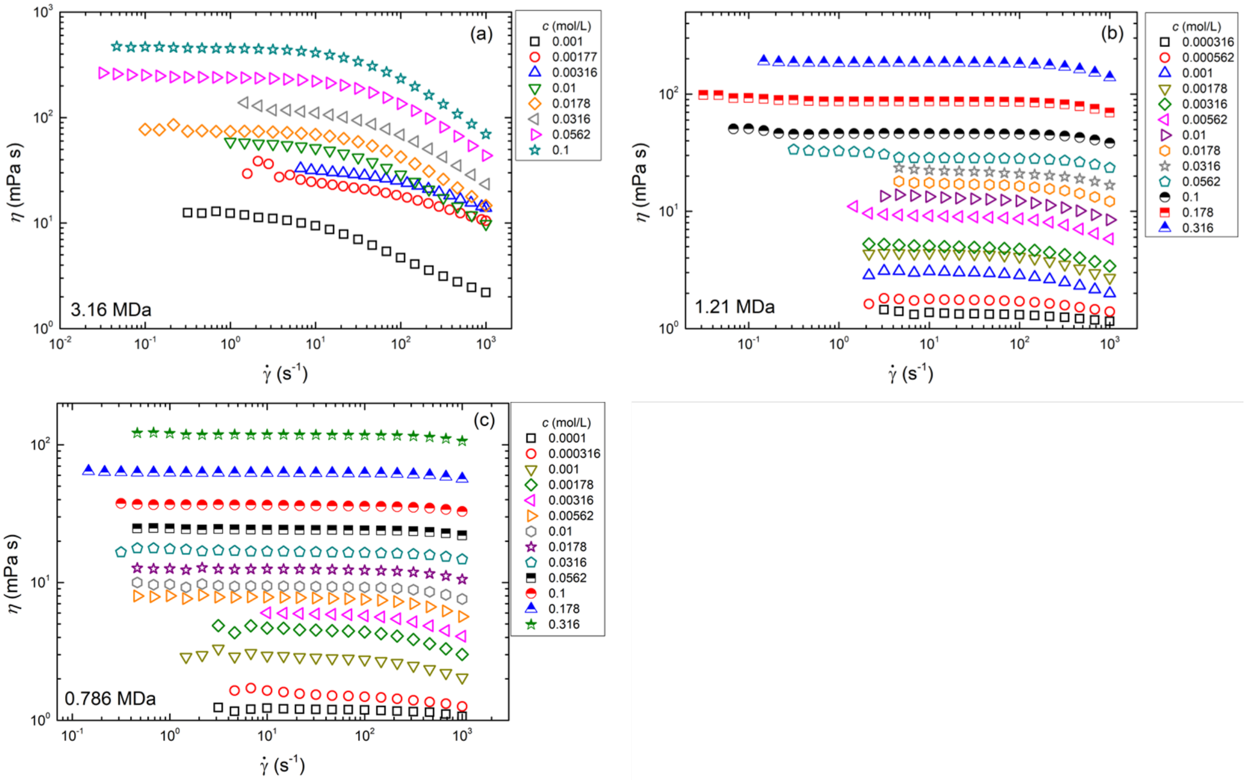

Figure S1. The shear viscosity curves for aqueous solutions of NaPSS with molecular weights of (a) 3.16 MDa, (b) 1.21 MDa, and (c) 0.786 MDa. Different symbols represent the shear viscosity curve of NaPSS solutions at various polymer concentrations.

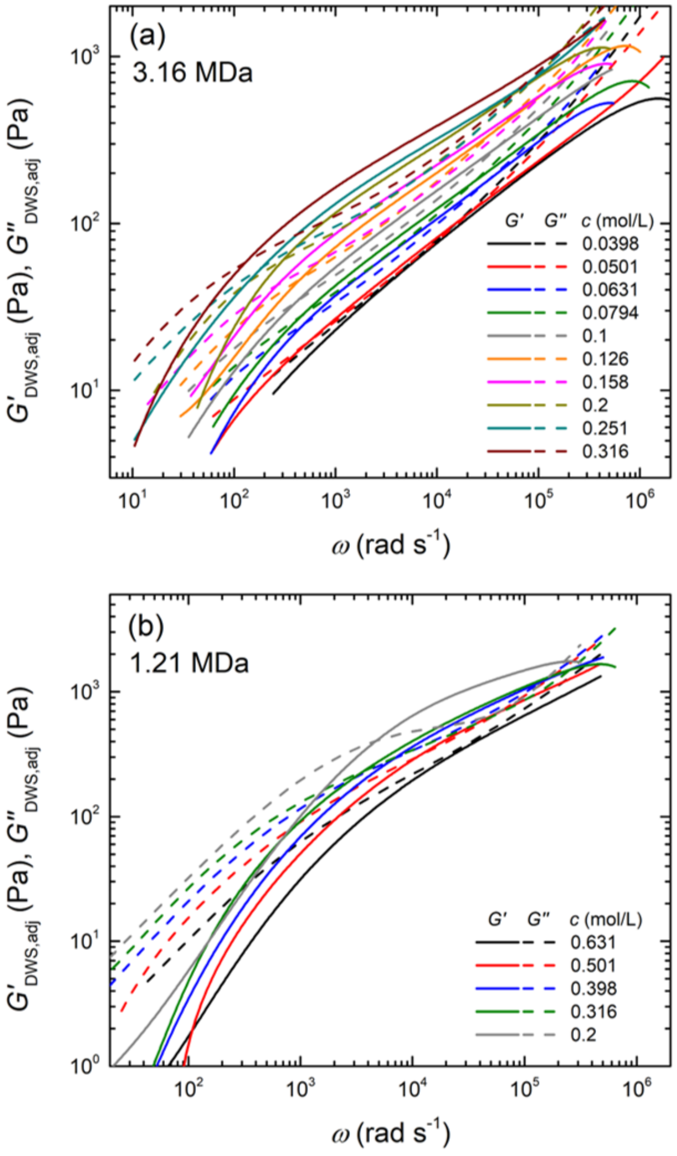

Figure S2. The dependence of the adjusted complex modulus $G^*_{\mathrm{DWS,adj}}$ on the angular frequency ω for aqueous solutions of two NaPSS with different molecular weights at (a) $M_\mathrm{w} = 3.16$ MDa and (b) $M_\mathrm{w} = 1.21$ MDa, obtained by the DWS measurement while varying the monomer concentration, c, of NaPSS. Solid and dashed lines denote the storage and loss moduli, respectively.

Table S1. Entanglement parameters extracted from the G^*_{DWS} spectra for the tested NaPSS samples.

$M_{\mathrm{w}} = 0.786$ MDa				
c (mol/L)	G_{N} (kPa)	τ_{rep} (ms)	τ_{e} (μs)	$\tau_{\mathrm{rep}}/\tau_{\mathrm{e}}$
0.501	0.79 ± 0.042	0.0936 ± 0.0075	11.9 ± 2.65	8.24 ± 2.17
0.631	1.32 ± 0.043	0.135 ± 0.007	12.2 ± 4.28	10.2 ± 2.44
0.794	1.95 ± 0.030	0.211 ± 0.022	7.78 ± 1.68	34.3 ± 13.8
1	3.12 ± 0.095	0.291 ± 0.023	9.56 ± 4.00	35.8 ± 18.9
$M_{\mathrm{w}} = 1.21$ MDa				
c (mol/L)	G_{N} (kPa)	τ_{rep} (ms)	τ_{e} (μs)	$\tau_{\mathrm{rep}}/\tau_{\mathrm{e}}$
0.316	0.47 ± 0.075	0.0859 ± 0.0143	17.4 ± 4.44	5.29 ± 2.09
0.398	0.61 ± 0.097	0.143 ± 0.007	6.81 ± 2.57	23.4 ± 8.54
0.501	0.64 ± 0.126	0.267 ± 0.062	13.4 ± 4.25	21.0 ± 5.79
0.631	1.17 ± 0.176	0.213 ± 0.064	4.42 ± 1.33	48.1 ± 14.4
$M_{\mathrm{w}} = 2.24$ MDa				
c (mol/L)	G_{N} (kPa)	τ_{rep} (ms)	τ_{e} (μs)	$\tau_{\mathrm{rep}}/\tau_{\mathrm{e}}$
0.1	0.11 ± 0.018	0.661 ± 0.077	18.0 ± 2.83	37.3 ± 7.80
0.126	0.16 ± 0.029	0.625 ± 0.188	15.9 ± 4.76	39.4 ± 11.8
0.158	0.17 ± 0.030	0.671 ± 0.154	16.4 ± 5.78	45.7 ± 20.4
0.2	0.21 ± 0.004	0.834 ± 0.129	10.8 ± 2.35	79.8 ± 21.9
0.251	0.24 ± 0.010	0.819 ± 0.176	8.16 ± 2.60	102 ± 10.9
0.316	0.44 ± 0.039	1.12 ± 0.278	13.0 ± 4.77	96.2 ± 57.9
0.398	0.59 ± 0.060	1.35 ± 0.069	7.97 ± 2.29	179 ± 46.9
$M_{\mathrm{w}} = 3.16$ MDa				
c (mol/L)	G_{N} (kPa)	τ_{rep} (ms)	τ_{e} (μs)	$\tau_{\mathrm{rep}}/\tau_{\mathrm{e}}$
0.0501	0.051 ± 0.003	1.69 ± 0.359	40.5 ± 9.58	45.0 ± 21.1
0.0631	0.052 ± 0.006	2.26 ± 0.609	38.5 ± 9.97	64.1 ± 32.0
0.0794	0.065 ± 0.004	1.52 ± 0.400	73.0 ± 25.2	21.8 ± 5.89
0.1	0.105 ± 0.008	1.97 ± 0.568	22.8 ± 7.17	96.8 ± 50.9
0.126	0.132 ± 0.016	1.91 ± 0.433	29.0 ± 1.79	66.2 ± 19.0
0.158	0.138 ± 0.015	4.32 ± 0.655	9.57 ± 2.75	480 ± 137
0.2	0.180 ± 0.014	3.25 ± 0.493	6.35 ± 1.48	528 ± 134
0.251	0.224 ± 0.011	4.47 ± 0.747	10.9 ± 2.90	438 ± 149
0.316	0.271 ± 0.011	7.17 ± 0.426	7.20 ± 3.18	1100 ± 303
